# Telaprevir combination therapy in HCV/HIV co-infected patients (INSIGHT study): sustained virologic response at 12 weeks final analysis

**DOI:** 10.7448/IAS.17.4.19626

**Published:** 2014-11-02

**Authors:** Marisa Montes, Mark Nelson, Pierre Marie Girard, Joe Sasadeusz, Andrzej Horban, Beatriz Grinsztejn, Natalia Zakharova, Antonio Rivero, Erkki Lathouwers, Katrien Janssen, Sivi Ouwerkerk-Mahadevan, James Witek

**Affiliations:** 1 HIV Unit, Internal Medicine, Hospital La Paz, Universidad Autónoma de Madrid, IdiPAZ, Madrid, Spain; 2 Chelsea and Westminster Hospital, London, UK; 3 Hôpital St Antoine, Paris, France; 4 Royal Melbourne and Alfred Hospitals, Melbourne, Australia; 5 Hospital of Infectious Diseases, Warsaw Medical University, Warsaw, Poland; 6 STD/AIDS Clinical Research Laboratory, Instituto de Pesquisa Clínica Evandro Chagas, Rio de Janeiro, Brazil; 7 Saint-Petersburg AIDS Center, St Petersburg, Russian Federation; 8 Hospital Universitario Reina Sofía-IMIBIC, Cordoba, Spain; 9 Janssen Infectious Diseases BVBA, Beerse, Belgium; 10 Janssen Research & Development LLC, Titusville, USA

## Abstract

**Introduction:**

We report the SVR12 final analysis of a phase 3 study of telaprevir in combination with peginterferon (P)/ribavirin (R) in HCV-genotype 1, treatment-naïve and -experienced patients with HCV/HIV co-infection (INSIGHT).

**Materials and Methods:**

Patients receiving stable, suppressive HIV antiretroviral (ARV) therapy, containing atazanavir/ritonavir, efavirenz, darunavir/ritonavir, raltegravir, etravirine or rilpivirine, received telaprevir 750 mg q8h (1125 mg q8h if on efavirenz) plus P (180 µg once-weekly) and R (800 mg/day) for 12 weeks, followed by an additional 12 weeks (non-cirrhotic HCV treatment-naïve and relapse patients with extended rapid viral response [eRVR]) or 36 weeks (all others) of PR alone. Analysis was performed when all patients had completed the follow-up visit of 12 weeks after last planned dose.

**Results:**

One hundred sixty-two patients were enrolled and treated (65 efavirenz, 59 atazanavir/ritonavir, 17 darunavir/ritonavir, 17 raltegravir, 4 etravirine). Mean age was 45 years, 78% were male, 92% were Caucasian; mean CD4 count was 687 cells/mm^3^. Sixty four patients (40%) were HCV treatment-naïve and 98 (60%) were treatment experienced (29 relapsers, 18 partial responders and 51 null responders). 64% were subtype 1a. 30% had bridging fibrosis (17%) or cirrhosis (13%). 19% of patients discontinued telaprevir, including 9% due to an adverse event (AE), 8% reaching a virologic endpoint and 2% for other reasons (non compliance or not defined). Treatment responses are shown in Table 1. There were no HIV RNA breakthroughs. Most frequently reported (≥20% patients) AEs were pruritus 43%; fatigue 27%; rash 34%, anorectal events 30% and influenza-like illness (25%). Anemia was reported in 15% of patients; grade ≥3 haemoglobin decrease occurred in 2.5% of patients. 6% of patients experienced serious AEs.

**Conclusions:**

In this phase 3 study of HIV-infected, HCV treatment-naïve and -experienced patients, 49% achieved eRVR and 57% reached SVR12. In patients with an eRVR, SVR12 rates were >80%, irrespective of prior treatment history.

**Table 1:** HCV RNA viral responses (Snapshot)

Undetectable HCV RNA* , n (%)	Treatment naïve (N=64)		Prior relapser (N=29)		Prior partial responder (N=18)		Prior null responder (N=51)		Overall (N=162)	
Response by eRVR										
Week 4 and 12 (eRVR)	YES	NO	YES	NO	YES	NO	YES	NO	YES	NO
	37	27	14	15	10	8	19	32	80	82
SVR12 planned (<25 IU/mL)	31/37 (84)	10/27 (37)	13/14 (93)	5/15 (33)	10/10 (100)	3/8 (38)	16/19 (84)	5/32 (16)	70/80 (88)	23/82 (28)
Response by planned treatment duration										
Planned treatment duration (weeks)	24	48	24	48	48		48		24 or 48	
On-treatment virologic failure	2 (5)	12 (44)	0	1 (6)	5 (28)		21 (41)		41 (25)	
Relapse	2 (5)	2 (7)	0	1 (6)	0		3 (6)		8 (5)	
SVR12 planned (<25 IU/mL)	31/37 (84)	10/27 (37)	12/13 (92)	6/16 (38)	13 (72)		21 (41)		93 (57)	

* HPS COBAS^®^ Taqman^®^ (v2.0, Roche): lower limit of quantification of 25IU/mL, limit of detection of 15IU/mL (genotype 1).

